# Expression of recombinant Cap antigen of porcine circovirus type 2 in the chloroplast of *Chlamydomonas reinhardtii*

**DOI:** 10.3389/fpls.2025.1661360

**Published:** 2025-10-09

**Authors:** Yiran Song, Youhong Lin, Xiaying Cheng, Wei Li, Yihong Li, Zongqi Yang

**Affiliations:** Key Laboratory of Plant Secondary Metabolism and Regulation of Zhejiang Province, College of Life Sciences and Medicine, Zhejiang Sci-Tech University, Hangzhou, China

**Keywords:** porcine circovirus type 2, *ORF2* antigen gene, capsid protein, *Chlamydomonas reinhardtii*, chloroplast transformation

## Abstract

The capsid (Cap) protein encoded by the *ORF2* gene of porcine circovirus type 2 is the major immunogen for the development of vaccines and can effectively reduce the incidence of porcine circovirus-associated diseases. In order to explore an efficient expression pathway of the recombinant Cap protein, using the *Chlamydomonas reinhardtii* chloroplast as the expression platform of the Cap protein, *C. reinhardtii* chloroplast expression vector pCR02 of the optimized *ORF2* gene was constructed and transferred into the *C. reinhardtii* chloroplast using the biolistic bombardment method. After multiple rounds of resistance screening and culturing, PCR, RT-PCR, Western blotting, and ELISA were used to detect the *ORF2* gene and the expression of the Cap protein in *C. reinhardtii* transformants. The results of the study showed that the *ORF2* antigen gene had been correctly integrated into the specific site of the *C. reinhardtii* chloroplast genome, and the Cap protein was effectively expressed. These results suggested that the *C. reinhardtii* chloroplast is a potential platform for the production of recombinant antigen proteins and provided important support for exploring new production pathways of recombinant pharmaceutical proteins.

## 1 Introduction

Porcine circovirus type 2 (PCV2), a member of the *Circoviridae* family, is the main pathogen of post-weaning multisystemic wasting syndrome (PMWS). PCV2 is not only related to a series of diseases, such as porcine respiratory disease complex and porcine dermatitis, but also susceptible to co-infection with other pathogens (Segalés et al., 2004; Opriessnig et al., 2007; Ramamoorthy and Meng, 2009; Alarcon et al., 2014; Lv et al., 2014), which has caused great economic losses to the swine industry around the world (Gillespie et al., 2009; Chi et al., 2014; Zhai et al., 2014). Since management strategies and co-infection control show limited efficacy in reducing porcine circovirus-associated disease (PCVAD) incidence, vaccination remains the most effective approach for preventing PCV2 infection (Chae, 2012). The immunogenic capsid (Cap) protein encoded by the *ORF2* gene is the target antigen of commercial vaccines due to its multiple antigenic epitopes (Cheung, 2003; Khayat et al., 2011; Zhang et al., 2015; Gava et al., 2018). Recombinant Cap proteins have been expressed in the *Escherichia coli* expression system (Wu et al., 2016), the yeast expression system (Bucarey et al., 2009; Tu et al., 2013), and the baculovirus/insect cell expression system (Liu et al., 2015; López-Vidal et al., 2015), and several baculovirus/insect cell-expressed proteins have successfully entered the marketing stage (Chae, 2012). However, the high production costs remain a significant bottleneck in expanding the application of the Cap antigen protein (Alarcon et al., 2014; Chi et al., 2014).

In recent years, the expression of pharmaceutical proteins in plant chloroplasts has become a research hotspot in plant biotechnology. Compared with the nucleus expression system, the chloroplast expression system has some unique advantages. For example, there is no gene silencing phenomenon and mechanism that inhibits the expression of recombinant proteins (Bock, 2007); the exogenous genes do not escape with the transmission of pollen, which contributes to improving bio-safety (Daniell, 2007). *Chlamydomonas reinhardtii*, also known as the “green yeast”, is the lower model plant in chloroplast genetic transformation research (Rochaix, 1995). Its growth cycle is short and culture is simple, which greatly reduces the production costs (Rochaix, 1995; Specht et al., 2010; Rasala and Mayfield, 2015). In addition, *C. reinhardtii* has a single large chloroplast, which contains many molecular chaperones and foldable enzymes (disulfide isomerase, proline isomerase, etc.) and provides a certain guarantee for the correct folding and assembly of recombinant proteins (Tran et al., 2013). At present, several pharmaceutical proteins have been successfully expressed in *C. reinhardtii* chloroplasts. In 2015, Barrera et al. (2015) expressed antitoxin drugs containing different domains of camel V_H_H antitoxin that can neutralize botulinum neurotoxin; in 2016, Ochoa-Méndez et al. (2016) constructed the expression vector of an anti-hypertensive drug (AHD) and successfully expressed it in the *C. reinhardtii* chloroplast; feeding results in mice with severe hypertension also showed that the drug can significantly decrease blood pressure. However, to date, there are a few reports on recombinant proteins expressed in the algal chloroplast (You-hong et al., 2017). It is unclear if this is due to a few attempts or to limitations of the system that preclude the expression of many proteins. In this study, we transformed the optimized *ORF2* gene of PCV2 into the chloroplast genome of *C. reinhardtii* by biolistic bombardment and expressed it to assess the capacity of transgenic algae as a recombinant protein production platform.

## 2 Materials and methods

### 2.1 Algal strains and materials

The wild-type *C. reinhardtii* strain is CC-137. Wild-type strains were cultured in 50 mL liquid Tris–acetate–phosphate (TAP) medium at 160 rpm and 25 °C under a photoperiod of 12 h/12 h (Harris, 1989). The precipitates were collected from 1.5 mL algal fluid of the logarithmic growth stage (3,000 rpm, 5 min), then suspended and coated on solid medium, and used for biolistic bombardment transformation after being cultured for 3–4 days.

Plasmids patpX (approximately 3.95 kb) and p64D (approximately 9.8 kb) were preserved in our laboratory. patpX contains a group of polyclonal sites and carries the 5′ promoter (including 5′-UTR) of the *atpA* gene and the 3′ terminator of the *rbcL* gene, which were the *C. reinhardtii* chloroplast genomic endogenous genes. Plasmid p64D contains the *aadA* resistance gene cassette and the homologous recombinant fragment (*clpP*–*trnL*–*petB*–*chlL*–*rpl23*–*rpl2*).

### 2.2 Optimization and synthesis of *ORF2* gene

In order to improve the expression efficiency of the major antigen gene-*ORF2* (GenBank: AY035820.1) of PCV2, the *ORF2* gene sequence was optimized and synthesized according to the preference of the chloroplast genome of *C. reinhardtii* by GenScript (Nanjing) Co., Ltd, Nanjing China. The *Nco*I and *Xba*I restriction sites were added at the 5′- and 3′-terminals of the codon-optimized *ORF2* sequence, respectively, to facilitate the construction of a chloroplast expression vector.

### 2.3 Construction of *C. reinhardtii* chloroplast expression vector

Plasmid patpX and the optimized *ORF2* gene were both excised by digestion with *Nco*I and *Xba*I, and the *ORF2* gene was ligated to the large fragment of patpX. The *ORF2* coding region was located between the chloroplast-specific promoter P*atpA*, 5’-UTR of *atpA* and the terminator of *rbcL*. *ORF2* gene expression cassette was obtained from the chloroplast expression intermediate vector pAF25. Plasmid pAF25 was excised by digestion with *Eco*RV and *Not*I, and then the smaller fragment (approximately 1.8 kb) was blunted with the Klenow large fragment enzyme and subcloned into p64D plasmid, which was excised with *Eco*RV. The specific *C. reinhardtii* chloroplast expression vector pCR02 of the *ORF2* gene was constructed.

### 2.4 *C. reinhardtii* chloroplast transformation and resistance screening

Gold particles coated with plasmid pCR02 were bombarded into the wild-type *C. reinhardtii* using the biolistic device PDS100/He (Bio-Rad, California, USA) (Kindle et al., 1991). After being cultured in darkness for 12 h, the bombarded *C. reinhardtii* cells were washed with TAP liquid medium and distributed on TAP solid medium containing 100 μg/mL spectinomycin. Under the conditions of a photoperiod of 12 h/12 h and 25 °C for approximately 10–14 days, the cells of *C. reinhardtii* without resistance gradually whitened and died, and the resistant cells grew into single green colonies on the solid selective medium. The single colonies of *C. reinhardtii* were selected and cultured in liquid selective medium (containing 100 μg/mL spectinomycin) for PCR analysis.

### 2.5 PCR detection of resistant algal cells

The total DNA of resistant algal cells and the wild-type strain was extracted using the cetyltrimethylammonium bromide (CTAB) method. Specific primers P1 (5′-CCATGGCTATGACTTATCCAC-3′) and P2 (5′-TCTAGATTATTTTGGATTTAATGGT-3′) were designed to detect the *ORF2* gene in resistant algal cells. Plasmid pCR02 was used as the positive control and wild-type *C. reinhardtii* as the negative control. The PCR procedures were as follows: 98 °C 3 min, 98 °C 10 s, 50 °C 10 s, 72 °C 15 s, and 72 °C 2 min, 35 cycles.

In addition, the integration site of the *ORF2* gene cassette in the *C. reinhardtii* chloroplast genome was detected using primers P3 (5′-CCGAACAATGTTTTTATTCCTGGAG-3′) and P4 (5′-TTCGAAAGCTGTACCTAAACCTACA-3′), which are complementary to the *ORF2* gene and *chlL* gene of the chloroplast genome of *C. reinhardtii*, respectively. Plasmid pCR02 was used as the positive control and wild-type *C. reinhardtii* as the negative control. PCR was performed as follows: 94 °C 5 min, 94 °C 30 s, 55 °C 30 s, 72 °C 2 min, and 72 °C 5 min, 35 cycles.

### 2.6 RT-PCR analysis of the transgenic algal cells

The total RNA of transgenic algal cells and wild-type *C. reinhardtii* was prepared to detect the transcription level of the *ORF2* gene using a total RNA extraction kit [Tiangen Biotech (Beijing) Co., Ltd., Beijing, China]. Total RNA (1 μg) was used to produce the cDNA of each line with the PrimeScript™ RT reagent kit with gDNA Eraser (Perfect Real Time, Takara, Dalian, China), and the RT-PCR detection of cDNA was performed using *ORF2*-specific primers P1 and P2 under the following conditions: 94 °C 5 min, 94 °C 30 s, 55 °C 30 s, 72 °C 1 min, and 72 °C 5 min, 35 cycles.

### 2.7 SDS–PAGE and Western blotting analysis

Crude proteins of *C. reinhardtii* transformants and the wild-type strain were extracted as described previously (Franklin et al., 2002). Liquid algal cells (15 mL) at the logarithmic growth phase were collected through centrifugation. The precipitate was suspended in 200 μL protein lysate (750 mM Tris–HCl, pH 8.0, 15% sucrose, 100 mM β-mercaptoethanol, and 1 mM Phenylmethanesulfonyl Fluoride (PMSF)) and centrifuged for 20 min at 12,000 r/min and 4 °C. The supernatants were composed of total soluble protein (TSP) and used for Cap protein analysis. Protein concentrations were determined using the Bradford protein assay. Approximately 10 μg total soluble protein from each sample was boiled for 5 min and loaded in the wells of a 12% polyacrylamide gel.

Protein samples were separated by 12% sodium dodecyl sulphate-polyacrylamide gel electrophoresis (SDS–PAGE) and directly transferred onto a polyvinylidene fluoride (PVDF) membrane (300 mA, 1 h). The membrane was then saturated in 5% skim milk powder for 2 h. After washing with tris-buffered saline with tween 20 (TBST), the membrane was successively incubated with rabbit anti-PCV2 Cap polyclonal antibody (1:10,000) (Beijing Biosynthesis Biotechnology Co., Ltd., Beijing, China) at 4 °C overnight and then incubated with 1:5,000 diluted goat anti-rabbit IgG antibody for 1–2 h at room temperature. The target protein was visualized by adding Enhanced Chemiluminescence (ECL) chemiluminescence chromogenic solution (Yeasen Biotech Co., Ltd., Shanghai China).

### 2.8 ELISA analysis

The antigenicity of the Cap protein in the crude extract of *C. reinhardtii* transformants was determined using ELISA. The extraction of protein samples was the same as above. Crude protein samples were added to a 96-well microtiter plate that had been coated with PCV2 Cap antibody to react at 37 °C for 30 min, washed with PBST, and treated with Horseradish Peroxidase (HRP)–PCV2 Cap antibody at 37 °C for 30 min. Then, the color solution was added and incubated at 37 °C for 15 min, and the absorbance was monitored at A450 after termination of the color reaction.

## 3 Results

### 3.1 Construction of the *C. reinhardtii* chloroplast expression vector

**Figure 1:**
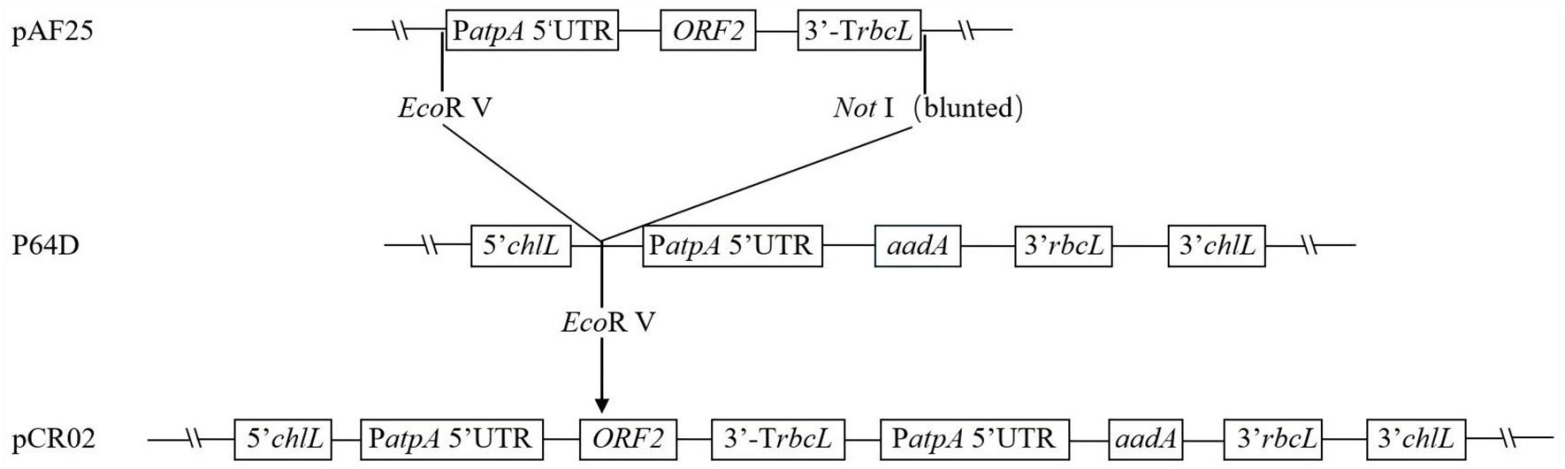
Construction of chloroplast expression vector pCR02. The pCR02 vector contains the optimized *ORF2* antigen gene cassette, *aadA* resistance gene cassette, and the homologous fragment (*clpP*–*trnL*–*petB*–*chlL*–*rpl23*–*rpl2*). The *ORF2* antigen gene is flanked by *Chlamydomonas reinhardtii* chloroplast-specific regulatory elements P*atpA* and T*rbcL*.

The *ORF2* gene sequence was optimized by GenScript (Nanjing) Co., Ltd., according to the preference of chloroplast gene expression in *C. reinhardtii*. A fragment of the *ORF2* gene expression cassette was excised from pAF25 with *Eco*RV and *Not*I, blunted with the Klenow large fragment enzyme, and then inserted into the *Eco*RV site of vector p64D to construct *C. reinhardtii* chloroplast expression vector pCR02. The construction process is shown in **Figure 1**.

### 3.2 Acquisition of resistant algal cells

**Figure 2:**
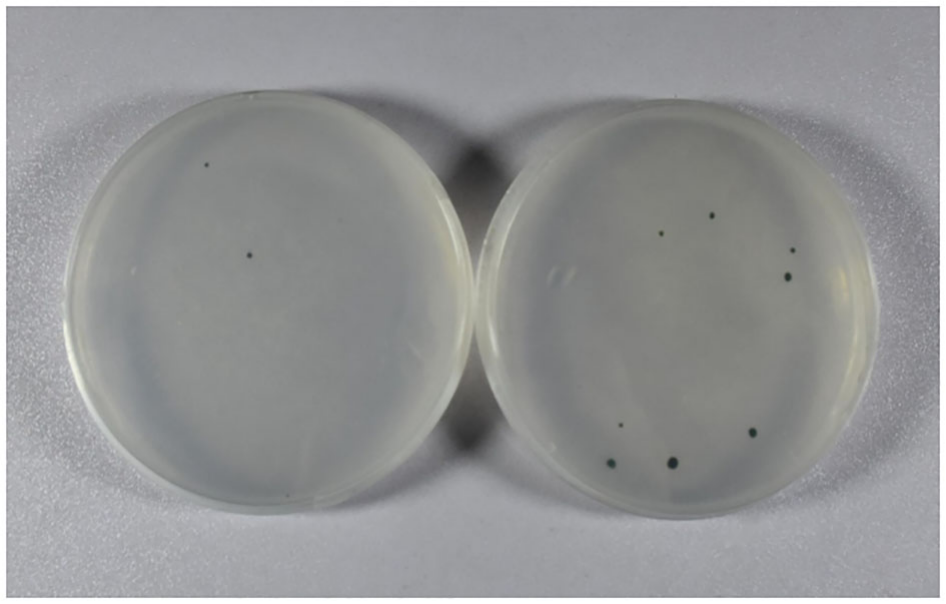
Acquisition of resistant algal cells. Resistant green algal colonies were obtained using the TAP solid medium containing 100 μg/mL spectinomycin. The other three resistant green algal colonies are not shown. TAP, Tris–acetate–phosphate.

Wild-type *C. reinhardtii* cells were bombarded with gold particles coated with plasmid pCR02 using the biolistic PDS1000/He (Bio-Rad) system (1,100 psi) and cultured on the TAP solid medium containing 100 μg/mL spectinomycin. After 10–14 days, 13 resistant green algal colonies appeared on the plates, while most algal cells were albino and died (**Figure 2**). This indicated that the *ORF2* gene and the *aadA* resistance gene may have been introduced into the chloroplast of *C. reinhardtii*, and the resistance gene has been expressed.

### 3.3 PCR analysis of resistant algal cells

**Figure 3:**
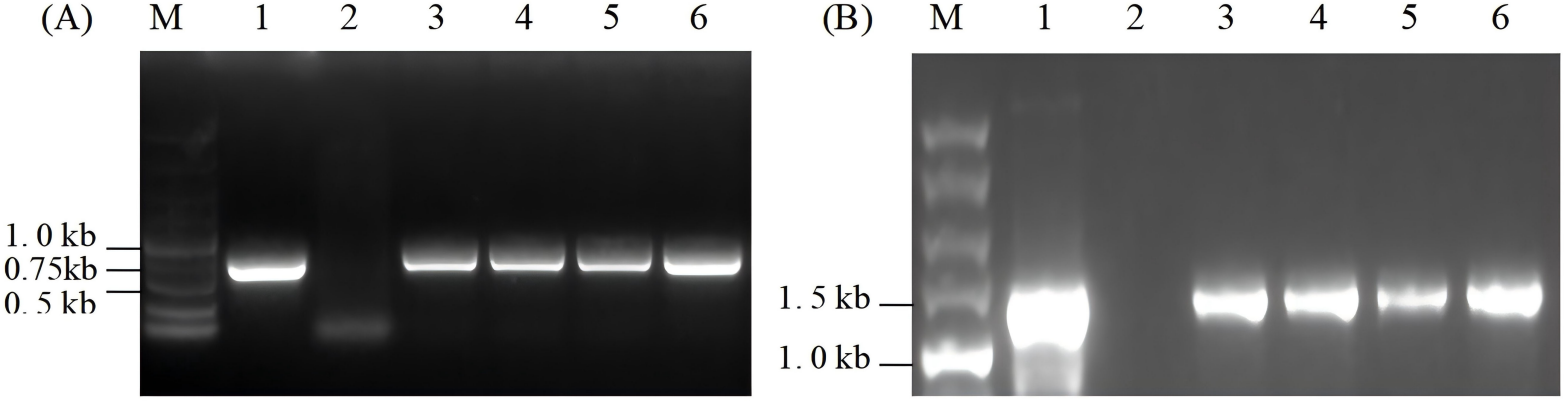
PCR analysis of resistant algal cells. **(A)** PCR detection of *ORF2* gene in resistant algal transformants. **(B)** Integration site analysis. Lane M, 5.0-kb DNA marker; Lane 1, the positive control; Lane 2, wild-type algal cells; Lanes 3–6, transformants of *Chlamydomonas reinhardtii*.

The total DNA of wild-type and resistant algal cells was extracted and detected using specific primers P1 and P2 of the *ORF2* gene. As shown in **Figure 3A**, the expected *ORF2* fragment, the 0.75-kb PCR product, was detected in four resistant algal cells and the positive control, but not in the wild-type algal strain. This indicated that the *ORF2* gene may be integrated into the chloroplast genome of *C. reinhardtii*.

To verify whether the *ORF2* gene was correctly integrated into the specific site of the chloroplast genome, primers P3 and P4, which are complementary to the chloroplast *chlL* gene and *ORF2* gene, respectively, were designed to detect the insertion site. Approximately 1.4-kb products were expected in algal transformants and the positive control, but no corresponding size product appeared in the wild-type. As shown in **Figure 3B**, 1.4-kb fragments were obtained in algal transformants that had undergone seven rounds of spectinomycin resistance screening, but did not appear in wild-type algal cells. The results suggested that the exogenous *ORF2* gene had been site-specifically integrated into the chloroplast genome of *C. reinhardtii*.

### 3.4 Transcription level assay of the *ORF2* gene in transgenic *C. reinhardtii*

**Figure 4:**
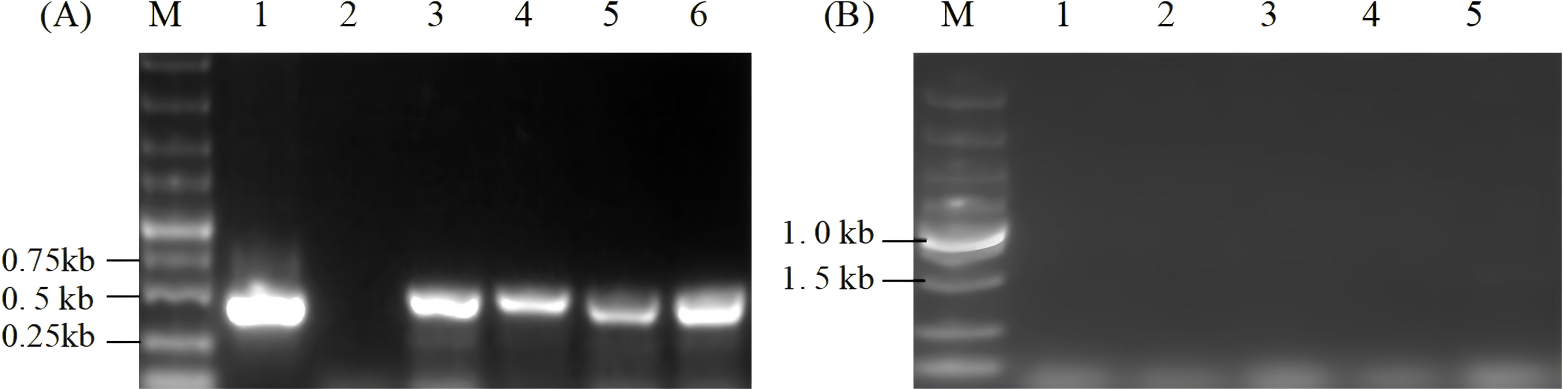
RT-PCR analysis of the *ORF2* gene in transgenic *Chlamydomonas reinhardtii* of resistant algal cells. **(A)** RT-PCR was performed using *ORF2*-specific primers P1 and P2. Lane M, 5.0-kb DNA markers; Lane 1, plasmid pCR02; Lane 2, cDNA of wild-type *C. reinhardtii*; Lanes 3–6, cDNA of independent transgenic *C. reinhardtii*. **(B)** RT-PCR analysis using total RNA without reverse transcription as templates. Lane M, 5-kb DNA marker; Lane 1, total RNA of wild-type *C. reinhardtii*; Lanes 2–5, total RNA of algal transformants.

The transcription level of the *ORF2* gene was determined through RT-PCR of the cDNA using specific primers P1 and P2 of the *ORF2* gene. As indicated in **Figure 4A**, the single 0.5-kb band in transgenic algal transformants displayed normal transcriptional status, whereas no band was amplified in wild-type algal cells. In addition, total RNA was directly used for PCR amplification to remove the samples that were possibly contaminated with DNA. The results showed that no bands were found in the same system and conditions, thus verifying the specificity of the RT-PCR (**Figure 4B**).

### 3.5 Western blotting analysis

**Figure 5:**
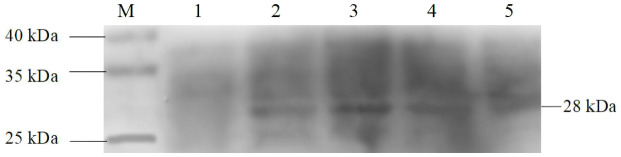
Western blotting analysis of the Cap protein in transgenic *Chlamydomonas reinhardtii*. Lane M, 10–250-kDa protein markers; Lane 1, wild-type *C. reinhardtii*; Lanes 2–5, *C. reinhardtii* transformants.

To verify the expression of the Cap protein in transgenic algal transformants, total soluble protein of transformants and wild-type *C. reinhardtii* were extracted and analyzed using SDS–PAGE and Western blotting. Rabbit anti-PCV2 Cap polyclonal antibody and goat anti-rabbit IgG antibody were used for hybridization. As shown in **Figure 5**, the 28-kDa protein was only detected in four *C. reinhardtii* transformants, but no protein was detected in the wild-type strain, which indicates that the Cap protein had accumulated in the chloroplast of *C. reinhardtii*.

### 3.6 ELISA analysis

**Figure 6:**
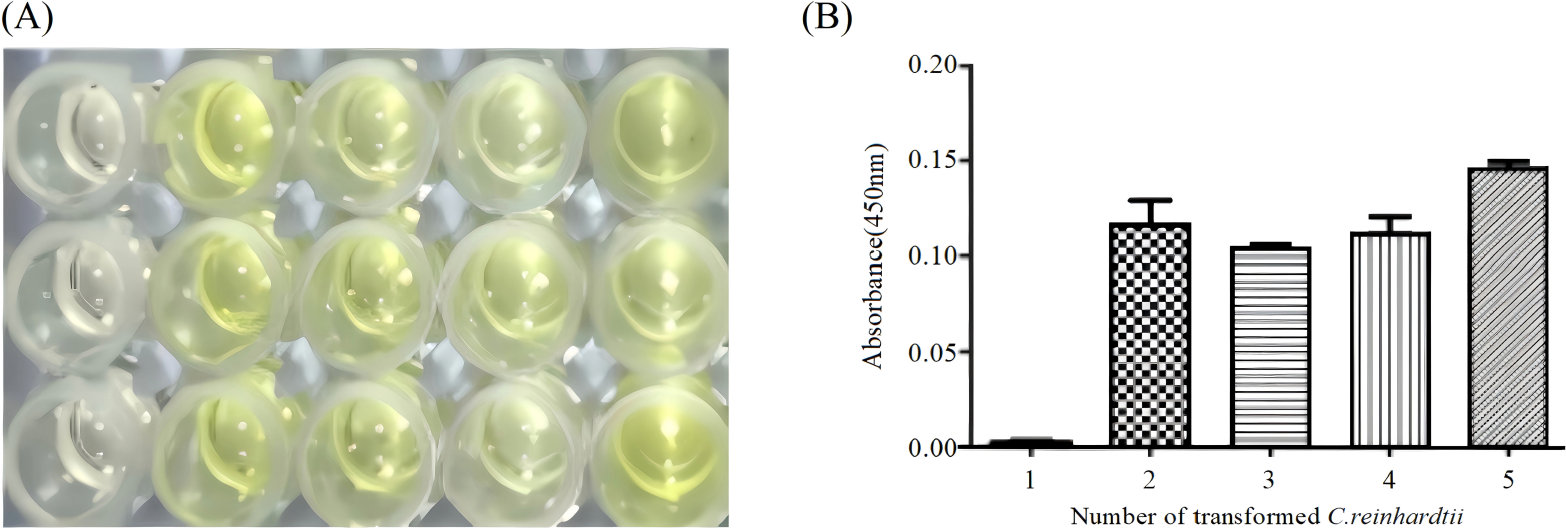
ELISA assay of Cap protein in *Chlamydomonas reinhardtii* transformants. **(A)** Color reaction of the Cap protein in four *C. reinhardtii* transformants and wild-type *C. reinhardtii*. Lane 1, wild-type *C. reinhardtii*; Lanes 2–5, *C. reinhardtii* transformants. **(B)** Absorbance of the Cap protein in four independent transgenic algal transformants and wild-type *C. reinhardtii*. Lane 1, wild-type *C. reinhardtii*; Lanes 2–5, *C. reinhardtii* transformants.

The antigenicity of the Cap protein expressed in *C. reinhardtii* chloroplasts was detected using ELISA. Total soluble protein samples extracted from wild-type and transgenic *C. reinhardtii* were reacted with PCV2 Cap antibody and HRP-conjugated PCV2 Cap antibody, and the absorbance was monitored at *OD*_450_. The results showed that significant color reactions were observed in four *C. reinhardtii* transformants (**Figure 6A**) and that absorption values were all detected (**Figure 6B**), indicating that the Cap protein expressed in *C. reinhardtii* chloroplasts had antigenicity.

## 4 Discussion

Although the Cap antigen vaccine expressed in the insect/baculovirus system is available, the production of genetically engineered vaccines in *C. reinhardtii* has received much attention recently due to several advantages: low production cost, the product enables oral delivery without purification, and both eukaryotic and prokaryotic proteins can be expressed in *C. reinhardtii* chloroplasts (Rasala and Mayfield, 2015). In this study, the optimized *ORF2* gene was recombined and expressed in the chloroplast of *C. reinhardtii*. The results showed that the Cap protein had been successfully expressed and had antigenicity, which proved that the *C. reinhardtii* chloroplast is a feasible expression platform for important recombinant proteins.

At present, the expression levels of exogenous proteins expressed in *Chlamydomonas* chloroplasts are generally low (Mayfield and Franklin, 2005; Liang et al., 2022; Jiang et al., 2025). The early-expressed β-glucuronidase (GUS) (Ishikura et al., 1999) and Renilla luciferase reporter proteins (Minko et al., 1999) in the chloroplasts of *C. reinhardtii* were almost undetectable in high endogenous protein backgrounds. There are several factors that may affect the expression of exogenous gene and protein accumulation in the *C. reinhardtii* chloroplast, including the promoter, 5′-UTR, and 3′-UTR, and optimization of the gene according to the preference of the chloroplast genome of *C. reinhardtii*. The transcription and expression of chloroplast genes are mainly determined using the promoter and 5′-UTR. Endogenous promoters from the *C. reinhardtii* chloroplast genome are required for the transcription and expression of exogenous genes (You-hong et al., 2017). The promoters of chloroplast endogenous genes, such as *atpA*, *psbA*, and *psbD*, were usually used to regulate the expression of foreign genes (Ishikura et al., 1999). Ishikura et al. (1999) expressed the *unid* gene in *C. reinhardtii* chloroplasts using the promoters of *rbcL*, *psbA*, and *atpA* genes and found that the *atpA* promoter had the best effect on the expression of the *unid* gene, while the promoter of *psbA* was almost ineffective. Michelet et al. (2011) found that the *psaA-exon*1 promoter was stronger than *atpA* and *psbA* in regulating the expression of *vap A* and *acr V* genes. The *atpA* promoter was examined and effectively regulated the transcription and expression of the *ORF2* gene in *C. reinhardtii* chloroplasts in this study. Other regulatory factors, such as 5′-UTRs of endogenous genes from the *C. reinhardtii* chloroplast genome, also affect the expression of foreign genes (Kasai et al., 2003; Barnes et al., 2005). Barnes et al. (2005) studied the effects of 5′-UTR and 3′-UTR of different genes on *gfp* gene expression and found that the 5′-UTR has a greater impact on the accumulation of exogenous proteins and mRNA than the 3′-UTR, which is almost ineffective on mRNA and protein accumulation. The 5′-UTR, including the promoter, of the plastid *atpA* and *psbD* genes produced the highest levels of chimeric mRNA and protein accumulation, while the 5′-UTR of the *rbcL* and *psbA* genes produced less mRNA and protein. Here, we show that the 5′-UTR of the *atpA* gene fusing with the *atpA* promoter can effectively drive mRNA and protein accumulation in *C. reinhardtii* chloroplasts. The *C. reinhardtii* chloroplast genome shows a high adenine and thymidine content as much as 66.3% and 80% of the third nucleotide of the codon including adenine or thymidine. It was reported that the expression level of GUS structural gene (*uidA*) (Ishikura et al., 1999) and Renilla luciferase reporter genes (Minko et al., 1999) in *Chlamydomonas* chloroplasts is almost undetectable under a high endogenous protein background. Mayfield et al. (Franklin and Mayfield, 2004; Mayfield and Franklin, 2005) suggested that the preference for codons in the chloroplast genome of *C. reinhardtii* may be one of the reasons for the low expression of exogenous genes in *C. reinhardtii* chloroplasts. To achieve protein expression, the sequence of the *ORF2* gene was optimized according to the preference of the *C. reinhardtii* chloroplast genome, and the Cap protein was expressed effectively.

## Data Availability

The original contributions presented in the study are publiclyavailable at the Jianguo cloud, which include the original images and date of ELISA. https://www.jianguoyun.com/p/DYT3digQ1NPfDRjfi4oGIAA.

## References

[B1] Alarcon P. Wieland B. Mateus A. L. Dewberry C. (2014). Pig farmers’ perceptions, attitudes, influences and management of information in the decision-making process for disease control. Prev. Vet. Med. 116, 223–242. doi: 10.1016/j.prevetmed.2013.08.004, PMID: 24016600

[B2] Barnes D. Franklin S. Schultz J. Henry R. Brown E. Coragliotti A.. (2005). Contribution of 5’- and 3’-untranslated regions of plastid mRNAs to the expression of Chlamydomonas reinhardtii chloroplast genes. Mol. Genet. Genomics 274, 625–636. doi: 10.1007/s00438-005-0055-y, PMID: 16231149

[B3] Barrera D. J. Rosenberg J. N. Chiu J. G. Chang Y. N. Debatis M. Ngoi S. M.. (2015). Algal chloroplast produced camelid VH H antitoxins are capable of neutralizing botulinum neurotoxin. Plant Biotechnol. J. 13, 117–124. doi: 10.1111/pbi.12244, PMID: 25229405 PMC4620920

[B4] Bock R. (2007). Plastid biotechnology: prospects for herbicide and insect resistance, metabolic engineering and molecular farming. Curr. Opin. Biotechnol. 18, 100–106. doi: 10.1016/j.copbio.2006.12.001, PMID: 17169550

[B5] Bucarey S. A. Noriega J. Reyes P. Tapia C. Sáenz L. Zuñiga A.. (2009). The optimized capsid gene of porcine circovirus type 2 expressed in yeast forms virus-like particles and elicits antibody responses in mice fed with recombinant yeast extracts. Vaccine 27, 5781–5790. doi: 10.1016/j.vaccine.2009.07.061, PMID: 19664739

[B6] Chae C. (2012). Commercial porcine circovirus type 2 vaccines: efficacy and clinical application. Vet. J. 194, 151–157. doi: 10.1016/j.tvjl.2012.06.031, PMID: 22841450

[B7] Cheung A. K. (2003). Transcriptional analysis of porcine circovirus type 2. Virology 305, 168–180. doi: 10.1006/viro.2002.1733, PMID: 12504550

[B8] Chi J. N. Wu C. Y. Chien M. S. Wu P. C. Wu C. M. Huang C. (2014). The preparation of porcine circovirus type 2 (PCV2) virus-like particles using a recombinant pseudorabies virus and its application to vaccine development. J. Biotechnol. 181, 12–19. doi: 10.1016/j.jbiotec.2014.04.006, PMID: 24739460

[B9] Daniell H. (2007). Transgene containment by maternal inheritance: effective or elusive? Proc. Natl. Acad. Sci. U.S.A. 104, 6879–6880. doi: 10.1073/pnas.0702219104, PMID: 17440039 PMC1855423

[B10] Franklin S. E. Mayfield S. P. (2004). Prospects for molecular farming in the green alga Chlamydomonas. Curr. Opin. Plant Biol. 7, 159–165. doi: 10.1016/j.pbi.2004.01.012, PMID: 15003216

[B11] Franklin S. Ngo B. Efuet E. Mayfield S. P. (2002). Development of a GFP reporter gene for Chlamydomonas reinhardtii chloroplast. Plant J. 30, 733–744. doi: 10.1046/j.1365-313x.2002.01319.x, PMID: 12061904

[B12] Gava D. Serrão V. H. B. Fernandes L. T. Cantão M. E. Ciacci-Zanella J. R. Morés N.. (2018). Structure analysis of capsid protein of Porcine circovirus type 2 from pigs with systemic disease. Braz. J. Microbiol. 49, 351–357. doi: 10.1016/j.bjm.2017.08.007, PMID: 29128395 PMC5914143

[B13] Gillespie J. Opriessnig T. Meng X. J. Pelzer K. Buechner-Maxwell V. (2009). Porcine circovirus type 2 and porcine circovirus-associated disease. J. Vet. Intern. Med. 23, 1151–1163. doi: 10.1111/j.1939-1676.2009.0389.x, PMID: 19780932 PMC7166794

[B14] Harris E. H. (1989). “The chlamydomonas sourcebook,” in A Comprehensive Guide to Biology and Laboratory Use, vol. 246 . Ed. Harris E. H. (Academic Press, San Diego, CA), 31–52. doi: 10.1126/science.246.4936.1503-a, PMID: 17756009

[B15] Ishikura K. Takaoka Y. Kato K. Sekine M. Yoshida K. Shinmyo A. (1999). Expression of a foreign gene in Chlamydomonas reinhardtii chloroplast. J. Biosci. Bioeng 87, 307–314. doi: 10.1016/s1389-1723(99)80037-1, PMID: 16232473

[B16] Jiang Y. Zhang G. Wang S. Raza A. Liu X. Guo C.. (2025). Expressing exogenous gene in Chlamydomonas reinhardtii chloroplast with viral replication elements. Bioresour Technol. 434, 132784. doi: 10.1016/j.biortech.2025.132784, PMID: 40490158

[B17] Kasai S. Yoshimura S. Ishikura K. Takaoka Y. Kobayashi K. Kato K.. (2003). Effect of coding regions on chloroplast gene expression in Chlamydomonas reinhardtii. J. Biosci. Bioeng 95, 276–282. doi: 10.1016/s1389-1723(03)80029-4, PMID: 16233405

[B18] Khayat R. Brunn N. Speir J. A. Hardham J. M. Ankenbauer R. G. Schneemann A.. (2011). The 2.3-angstrom structure of porcine circovirus 2. J. Virol. 85, 7856–7862. doi: 10.1128/jvi.00737-11, PMID: 21632760 PMC3147897

[B19] Kindle K. L. Richards K. L. Stern D. B. (1991). Engineering the chloroplast genome: techniques and capabilities for chloroplast transformation in Chlamydomonas reinhardtii. Proc. Natl. Acad. Sci. U.S.A. 88, 1721–1725. doi: 10.1073/pnas.88.5.1721, PMID: 11607155 PMC51096

[B20] Liang W. Qiu J. Zhang M. Wang C. (2022). Heterologous expression of human C-reactive protein in the green alga Chlamydomonas reinhardtii. J. Food Biochem. 46, e14067. doi: 10.1111/jfbc.14067, PMID: 34981544

[B21] Liu Y. Zhang Y. Yao L. Hao H. Fu X. Yang Z.. (2015). Enhanced production of porcine circovirus type 2 (PCV2) virus-like particles in Sf9 cells by translational enhancers. Biotechnol. Lett. 37, 1765–1771. doi: 10.1007/s10529-015-1856-7, PMID: 25994579

[B22] López-Vidal J. Gómez-Sebastián S. Bárcena J. Nuñez Mdel C. Martínez-Alonso D. Dudognon B.. (2015). Improved production efficiency of virus-like particles by the baculovirus expression vector system. PloS One 10, e0140039. doi: 10.1371/journal.pone.0140039, PMID: 26458221 PMC4601761

[B23] Lv Q. Z. Guo K. K. Zhang Y. M. (2014). Current understanding of genomic DNA of porcine circovirus type 2. Virus Genes 49, 1–10. doi: 10.1007/s11262-014-1099-z, PMID: 25011695

[B24] Mayfield S. P. Franklin S. E. (2005). Expression of human antibodies in eukaryotic micro-algae. Vaccine 23, 1828–1832. doi: 10.1016/j.vaccine.2004.11.013, PMID: 15734050

[B25] Michelet L. Lefebvre-Legendre L. Burr S. E. Rochaix J. D. Goldschmidt-Clermont M. (2011). Enhanced chloroplast transgene expression in a nuclear mutant of Chlamydomonas. Plant Biotechnol. J. 9, 565–574. doi: 10.1111/j.1467-7652.2010.00564.x, PMID: 20809927

[B26] Minko I. Holloway S. P. Nikaido S. Carter M. Odom O. W. Johnson C. H.. (1999). Renilla luciferase as a vital reporter for chloroplast gene expression in Chlamydomonas. Mol. Gen. Genet. 262, 421–425. doi: 10.1007/s004380051101, PMID: 10589828

[B27] Ochoa-Méndez C. E. Lara-Hernández I. González L. M. Aguirre-Bañuelos P. Ibarra-Barajas M. Castro-Moreno P.. (2016). Bioactivity of an antihypertensive peptide expressed in Chlamydomonas reinhardtii. J. Biotechnol. 240, 76–84. doi: 10.1016/j.jbiotec.2016.11.001, PMID: 27816654

[B28] Opriessnig T. Meng X. J. Halbur P. G. (2007). Porcine circovirus type 2 associated disease: update on current terminology, clinical manifestations, pathogenesis, diagnosis, and intervention strategies. J. Vet. Diagn. Invest. 19, 591–615. doi: 10.1177/104063870701900601, PMID: 17998548

[B29] Ramamoorthy S. Meng X. J. (2009). Porcine circoviruses: a minuscule yet mammoth paradox. Anim. Health Res. Rev. 10, 1–20. doi: 10.1017/s1466252308001461, PMID: 18761774

[B30] Rasala B. A. Mayfield S. P. (2015). Photosynthetic biomanufacturing in green algae; production of recombinant proteins for industrial, nutritional, and medical uses. Photosynth Res. 123, 227–239. doi: 10.1007/s11120-014-9994-7, PMID: 24659086

[B31] Rochaix J. D. (1995). Chlamydomonas reinhardtii as the photosynthetic yeast. Annu. Rev. Genet. 29, 209–230. doi: 10.1146/annurev.ge.29.120195.001233, PMID: 8825474

[B32] Segalés J. Rosell C. Domingo M. (2004). Pathological findings associated with naturally acquired porcine circovirus type 2 associated disease. Vet. Microbiol. 98, 137–149. doi: 10.1016/j.vetmic.2003.10.006, PMID: 14741126

[B33] Specht E. Miyake-Stoner S. Mayfield S. (2010). Micro-algae come of age as a platform for recombinant protein production. Biotechnol. Lett. 32, 1373–1383. doi: 10.1007/s10529-010-0326-5, PMID: 20556634 PMC2941057

[B34] Tran M. Van C. Barrera D. J. Pettersson P. L. Peinado C. D. Bui J.. (2013). Production of unique immunotoxin cancer therapeutics in algal chloroplasts. Proc. Natl. Acad. Sci. U.S.A. 110, E15–E22. doi: 10.1073/pnas.1214638110, PMID: 23236148 PMC3538218

[B35] Tu Y. Wang Y. Wang G. Wu J. Liu Y. Wang S.. (2013). High-level expression and immunogenicity of a porcine circovirus type 2 capsid protein through codon optimization in Pichia pastoris. Appl. Microbiol. Biotechnol. 97, 2867–2875. doi: 10.1007/s00253-012-4540-z, PMID: 23143467

[B36] Wu P. C. Chen T. Y. Chi J. N. Chien M. S. Huang C. (2016). Efficient expression and purification of porcine circovirus type 2 virus-like particles in Escherichia coli. J. Biotechnol. 220, 78–85. doi: 10.1016/j.jbiotec.2016.01.017, PMID: 26795354

[B37] You-hong L. Xia-ying C. Yi-wen Y. Zong-suo L. Zong-qi Y. (2017). Expression and optimization strategy of RecombinantProteins in chlamydomonas chloroplast. China Biotechnol. 37, 118–125. doi: 10.13523/j.cb.20171016

[B38] Zhai S. L. Chen S. N. Xu Z. H. Tang M. H. Wang F. G. Li X. J.. (2014). Porcine circovirus type 2 in China: an update on and insights to its prevalence and control. Virol. J. 11, 88. doi: 10.1186/1743-422x-11-88, PMID: 24885983 PMC4031328

[B39] Zhang H. Qian P. Peng B. Shi L. Chen H. Li X. (2015). A novel subunit vaccine co-expressing GM-CSF and PCV2b Cap protein enhances protective immunity against porcine circovirus type 2 in piglets. Vaccine 33, 2449–2456. doi: 10.1016/j.vaccine.2015.03.090, PMID: 25863115

