# Supplementary material for: Expression of recombinant Cap antigen of porcine circovirus type 2 in the chloroplast of Chlamydomonas reinhardtii
Source: Front Plant Sci. 2025 Oct 9;16:1661360. doi: 10.3389/fpls.2025.1661360 (PMC12546175; doi:10.3389/fpls.2025.1661360)
Supplement: Supplementary file 1 [file DataSheet1.pdf]

Supplementary Material

1 Supplementary Figures

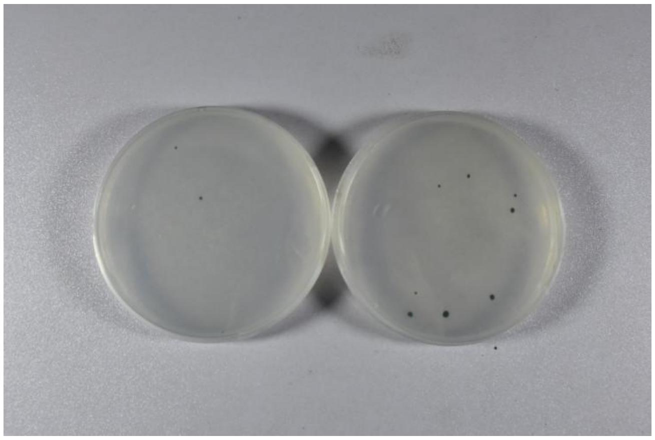

Original image of Figure 2

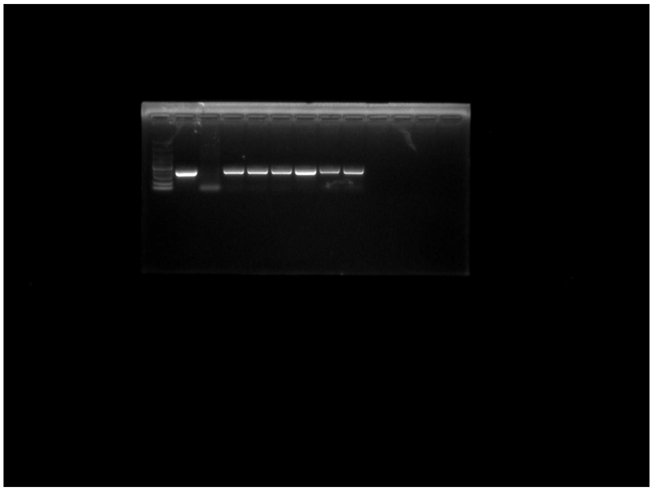

Original image of Figure 3A

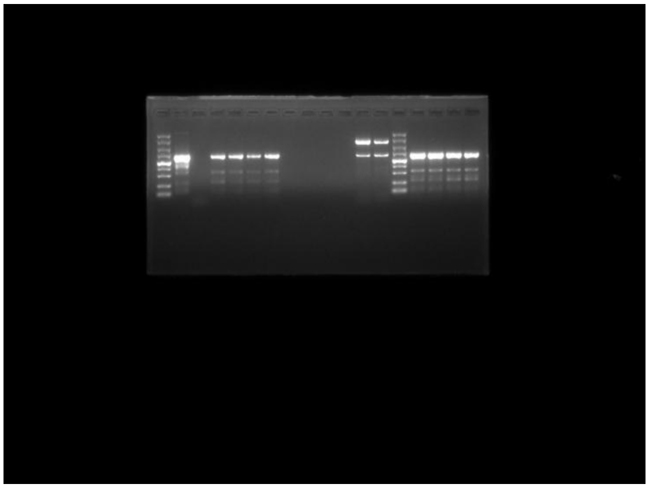

Original image of Figure 3B in left

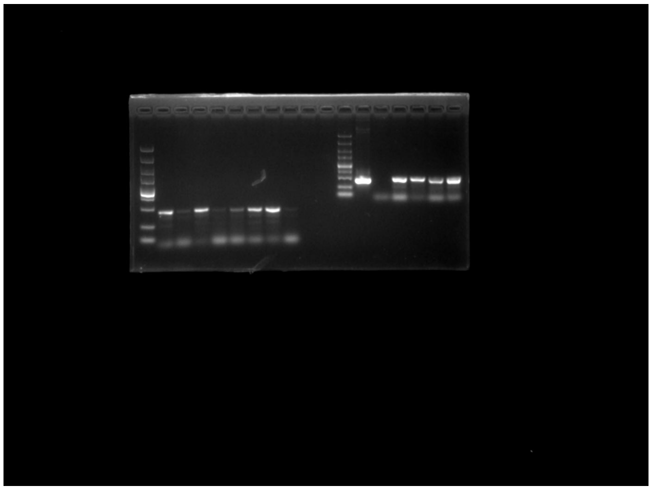

Original image of Figure 4A in right

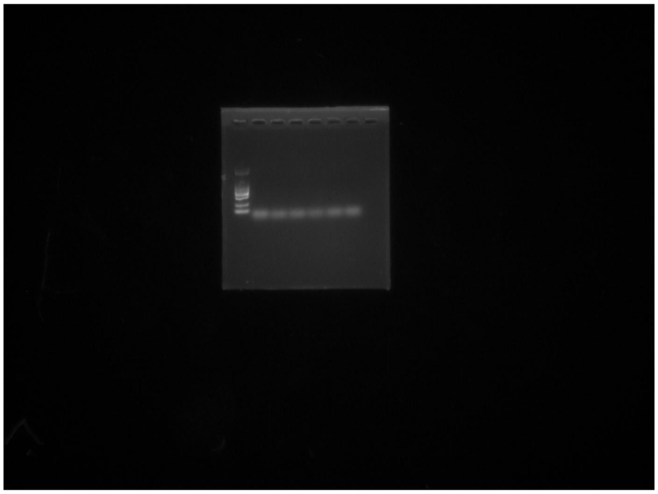

Original image of Figure 4B

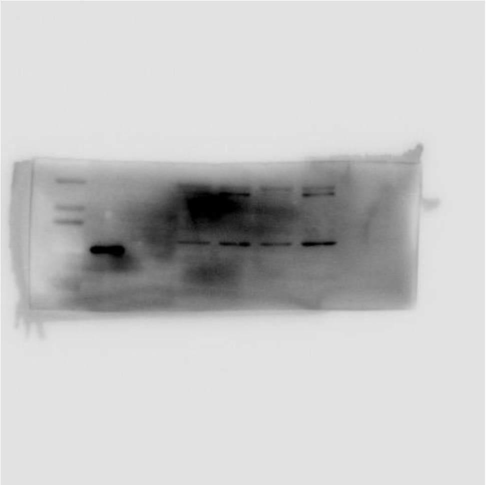

Original image of Figure 5
